# The Mitotic Arrest Deficient Protein MAD2B Interacts with the Clathrin Light Chain A during Mitosis

**DOI:** 10.1371/journal.pone.0015128

**Published:** 2010-11-30

**Authors:** Klaas Medendorp, Lilian Vreede, Jan J. M. van Groningen, Lisette Hetterschijt, Linda Brugmans, Patrick A. M. Jansen, Wilhelmina H. van den Hurk, Diederik R. H. de Bruijn, Ad Geurts van Kessel

**Affiliations:** 1 Department of Human Genetics, Radboud University Nijmegen Medical Centre, Nijmegen Centre for Molecular Life Sciences, Nijmegen, The Netherlands; 2 Department of Dermatology, Radboud University Nijmegen Medical Centre, Nijmegen Centre for Molecular Life Sciences, Nijmegen, The Netherlands; Queensland University of Technology, Australia

## Abstract

**Background:**

Although the mitotic arrest deficient protein MAD2B (MAD2L2) is thought to inhibit the anaphase promoting complex (APC) by binding to CDC20 and/or CDH1 (FZR1), its exact role in cell cycle control still remains to be established.

**Methodology/Principal Findings:**

Using a yeast two-hybrid interaction trap we identified the human clathrin light chain A (CLTA) as a novel MAD2B binding protein. A direct interaction was established in mammalian cells via GST pull-down and endogenous co-immunoprecipitation during the G2/M phase of the cell cycle. Through subsequent confocal laser scanning microscopy we found that MAD2B and CLTA co-localize at the mitotic spindle. Clathrin forms a trimeric structure, i.e., the clathrin triskelion, consisting of three heavy chains (CLTC), each with an associated light chain. This clathrin structure has previously been shown to be required for the function of the mitotic spindle through stabilization of kinetochore fibers. Upon siRNA-mediated MAD2B depletion, we found that CLTA was no longer concentrated at the mitotic spindle but, instead, diffusely distributed throughout the cell. In addition, we found a marked increase in the percentage of misaligned chromosomes.

**Conclusions/Significance:**

Previously, we identified MAD2B as an interactor of the renal cell carcinoma (RCC)-associated protein PRCC. In addition, we found that fusion of PRCC with the transcription factor TFE3 in t(X;1)(p11;q21)-positive RCCs results in an impairment of this interaction and a concomitant failure to shuttle MAD2B to the nucleus. Our current data show that MAD2B interacts with CLTA during the G2/M phase of the cell cycle and that depletion of MAD2B leads to a marked increase in the percentage of misaligned chromosomes and a redistribution of CLTA during mitosis.

## Introduction

The mitotic arrest deficient protein MAD2B (MAD2L2) is a member of a MAD family of proteins and shows 48% similarity to MAD2 (MAD2L1) [1], [2]. Both MAD2 and MAD2B can bind to the anaphase promoting complex or cyclosome (APC/C), which is a downstream target of the mitotic spindle checkpoint. APC/C is activated by CDC20 and/or CDH1 (FZR1), whereas MAD2 and MAD2B can act as APC/C inhibitors by binding to CDC20 and/or CDH1, respectively [3], [4]. More recently, it was found that during mitosis clathrin is localized to kinetochore fibres of the spindle [5]. Clathrin is composed of heavy (CLTC) and light (CLTA/CLTB) chains, respectively, and direct binding of clathrin to the kinetochore fibres is thought to be mediated by the heavy chain, since in the absence of CLTC, clathrin light chains are no longer recruited to the spindle [5]. Once bound to the mitotic spindle apparatus, clathrin stabilizes mitotic microtubules by forming a trimeric structure, i.e., the clathrin triskelion [6]. RNAi-mediated knock down of CLTC leads to kinetochore fiber de-stabilization and concomitant defective congression of chromosomes to the metaphase plate and persistent activation of the spindle checkpoint. Normally, MAD2 is localized to kinetochores in early prometaphase and becomes diffusely distributed in metaphase. Since in CLTC depleted cells MAD2 was found to be located at the kinetochores of both misaligned chromosomes and chromosomes at the metaphase plate, it was concluded that the persistent spindle checkpoint activation may be mediated by MAD2 signalling [5]. Together, these observations suggest that clathrin may act as an integral component of the mitotic spindle apparatus and that its abrogation may lead to chromosomal instability and, ultimately, cancer [7]–[9]. Interestingly, fusions of CLTC with the anaplastic lymphoma kinase (ALK) have been encountered in inflammatory myofibroblastic tumors and anaplastic large-cell lymphomas [10], whereas fusions with the transcription factor TFE3 have been encountered in renal cell carcinomas (RCC) [11], [12]. These fusions are expected to disrupt clathrin trimerization, thereby impairing its function during mitosis [5]. Previously, we found that in RCCs carrying a recurrent t(X;1)(p11;q21) chromosome translocation TFE3 is fused to a novel protein, PRCC [13], [14]. Subsequently, we found that PRCC can interact with MAD2B and, by doing so, mediate its shuttling to the nucleus [15]. The PRCCTFE3 fusion protein, however, has lost this capacity in spite of the fact that the PRCC interaction domain is retained in the fusion product. Based on these results, we previously hypothesized that the putative role of MAD2B in cell cycle control may be abrogated through PRCCTFE3 expression in t(X;1)(p11;q21)-positive RCCs [15]. To further assess this role, we performed a yeast two-hybrid interaction screen with MAD2B as a bait. By doing so, we identified the clathrin light chain CLTA as a novel *bona fide* interactor.

## Results

### Identification of CLTA as a MAD2B binding protein

**Figure 1:**
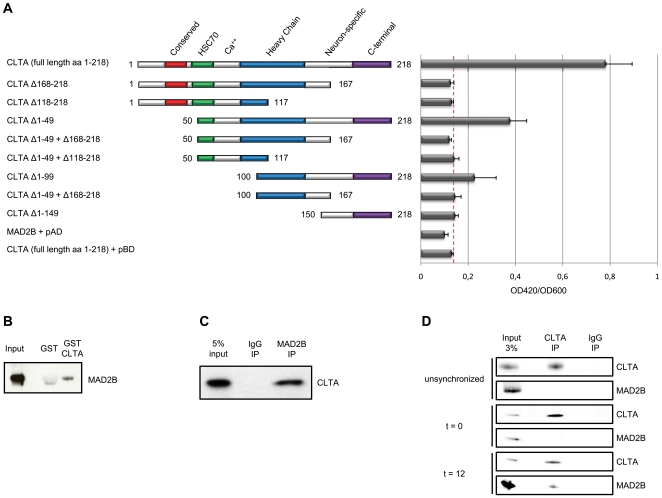
MAD2B and CLTA interact physically. (A) Schematic representation of the CLTA (deletion) constructs (numbers correspond to amino acid positions) and corresponding interactions with MAD2B measured as OD420/OD600 ratios using a liquid β-galactosidase assay. (B) HA-MAD2B protein was probed for interaction with either GST or GST-CLTA. Subsequent western blot analysis was performed using an anti-HA tag antibody. (C) U2OS cells were transiently transfected with HA-MAD2B and FLAG-CLTA. Immunoprecipitations (IP) were performed with a control rabbit antibody (IgG) and an anti-MAD2B antibody as indicated. Western blot analysis was performed using an anti-FLAG antibody. (D) U2OS cells were synchronized by a double thymidine block (see Materials and methods) and subsequently harvested at different time intervals after release from this cell cycle block. Immunoprecipitations were performed on cell lysates from unsynchronized cells and from cells at t = 0 and t = 12 after release. Endogenous proteins were precipitated with an anti-CLTA and a control mouse antibody (IgG) as indicated. Western blot analysis was performed using anti-CLTA and anti-MAD2B antibodies.

In order to further assess the role of MAD2B in cell cycle control, we set out to identify binding proteins using a yeast two-hybrid interaction trap (see Materials and methods). By employing the full coding sequence of MAD2B as bait and a human testis cDNA library as prey, we recovered 92 colonies that grew on fully selective SD medium. Of these colonies, 87 turned blue in a LacZ filter lift assay and 69 were selected for further analysis. The corresponding plasmids were recovered and re-introduced into the same yeast cells and, subsequently, these cells were plated in fully selective SD medium and re-screened for β-galactosidase activity. These combined assays yielded six independent plasmids, all encompassing coding sequences for the clathrin light chain A (CLTA) protein. Among these six clones, five clones contained full-length CLTA coding sequences and one clone truncated sequences coding for its C-terminal 100 amino acids. Co-transfection of these prey clones with standard control plasmids encoding lamin-B and p53 turned out to be negative, thereby validating the yeast two-hybrid interactions observed. In order to refine the identification of a specific domain of CLTA required for interacting with MAD2B, a series of deletion constructs was generated lacking consecutive stretches of 50 amino acids at either the C- or N-terminal regions of the coding sequence (Fig. 1A). The resulting CLTA (deletion) constructs were co-transfected with full-length MAD2B into yeast cells and tested for their ability to grow on fully selective SD medium and for β-galactosidase activity. The data obtained indicate that the C-terminal region (containing amino acids 151–218) is required for the MAD2B-CLTA interaction, since all C-terminal deletions of the CLTA protein resulted in a complete abrogation of yeast growth (not shown) and β-galactosidase activity (Fig. 1A). In addition, the central domain of CLTA appears to be essential, but not sufficient, for binding to the MAD2B protein. These observations are completely in line with our initial yeast two-hybrid screening data. A reduction of the critical interaction domain within the MAD2B protein could not be attained, since all deletions, including N-terminal and C-terminal deletions (see materials and methods), resulted in a complete abolition of the MAD2B-CLTA interaction (data not shown).

### MAD2B and CLTA interact physically

To investigate whether the MAD2B protein physically interacts with CLTA, we performed a glutathione S-transferase (GST) pull-down assay. To this end, full length GST-CLTA fusion protein was produced in *E. coli* cells, and full-length HA-tagged MAD2B (HA-MAD2B) was expressed in U2OS cells. After pull down of the GST-CLTA fusion protein, and GST as a control, the samples were analyzed by western blotting using an anti-HA antibody. By doing so, we found that the MAD2B protein (expected size, 28–29 kDa) [3] was pulled down with the GST-CLTA fusion protein, but not with GST alone (Fig. 1B), thus supporting a physical *in vitro* interaction between MAD2B and CLTA. To further confirm this interaction *in vivo*, U2OS cells were transiently transfected with HA-MAD2B and FLAG-tagged CLTA (FLAG-CLTA) expression constructs. Subsequently, immunoprecipitations were performed on the double transfectants using an anti-MAD2B antibody to precipitate MAD2B and/or HA-MAD2B. After western blotting using an anti-FLAG antibody, the FLAG-CLTA protein could readily be observed in the input and the anti-MAD2B immunoprecipitate, but not in the mock (IgG) immunoprecipitate (Fig. 1C), thus confirming the *in vivo* interaction between exogenous MAD2B and CLTA in mammalian cells. Subsequently, the endogenous interaction between MAD2B and CLTA was assessed. To this end, U2OS cells were synchronized (double thymidine block, see Materials and methods) and subsequently harvested at different time points after release from this cell cycle block. Concomitant FACS analyses (Fig. S1) revealed that the majority of unsynchronized cells was in the G0 and/or G1 phase of the cell cycle and that before release from the cell cycle block (t = 0) the majority of cells was in the S phase of the cell cycle, as expected. At 12 hours after release from the cell cycle block (t = 12) the majority of cells was in the G2 and/or M phase. After protein extraction from these respective samples, immunoprecipitations were carried out using an anti-CLTA antibody. Subsequent western blot analysis with an anti-MAD2B antibody readily revealed MAD2B protein in the CLTA immunoprecipitate at t = 12, but not in the other immunoprecipitation samples (i.e., unsynchronized and t = 0; Fig. 1D), thereby confirming an endogenous interaction of these two proteins, primarily during the G2 and/or M phase of the cell cycle.

Since CLTA is known to form a complex with the clathrin heavy chain (CLTC) during mitosis, i.e., the triskelion, we investigated whether endogenous MAD2B can bind to this complex. To this end, U2OS cells were grown in the presence of ^35^S labeled methionine and, subsequently, whole cell lysates were prepared and used for immunoprecipitation with an anti-MAD2B, an anti-CLTA and a control IgG antibody. By doing so, we observed a strong band of ∼190 kDa (CLTC; Fig. S2A) after immunoprecipitation with the anti-CLTA antibody, as expected [9], [16]. This same band was also observed after immunoprecipitation with the anti-MAD2B antibody, but not with the control IgG antibody, thus indicating that endogenous MAD2B can indeed bind to the CLTA-CLTC complex, presumably in its triskelion configuration.

### MAD2B and CLTA co-localize at the mitotic spindle

**Figure 2:**
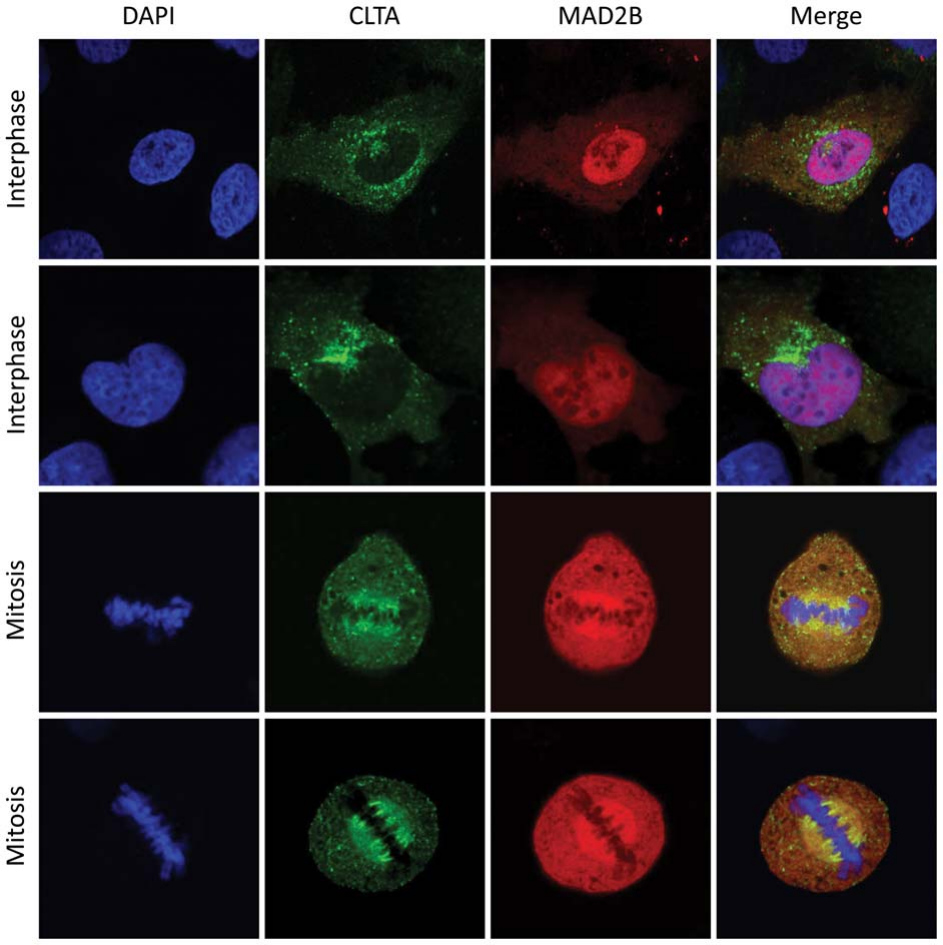
MAD2B and CLTA co-localize at the mitotic spindle. Cells were transiently transfected with CLTA-GFP (green) and MAD2B-mRFP (red) constructs. DAPI staining (blue) marks the position of the nuclei and chromosomes. Yellow staining in the overlay indicates co-localization of MAD2B and CLTA. The upper two panels show cells in interphase, the lower two panels show cells in mitosis.

In order to further assess the observed interaction between MAD2B and CLTA in mammalian cells, expression constructs carrying full-length coding sequences of mRFP-tagged MAD2B and GFP-tagged CLTA were transiently transfected into U2OS cells. After confocal laser scanning microscopy, we found that MAD2B exhibited a diffuse staining pattern in both the cytoplasm and the nucleus (Fig. 2, upper panels), as expected [15], [17]. Also as expected [5], [18], CLTA showed a punctate membranous/cytoplasmic distribution with an accumulation at the Golgi apparatus (Fig. 2, upper panels). CLTA was not observed within the nucleus during interphase. Accordingly, CLTA and MAD2B failed to show any overt sub-cellular co-localization during interphase. In order to assess the sub-cellular localization of both proteins during mitosis, U2OS cells were again synchronized with a double thymidine block (see above) and fixed at different time intervals after release. By doing so, we found that during mitosis CLTA was located at the mitotic spindle co-localizing with α-tubulin (Fig. S3). MAD2B exhibited a very similar localization at the mitotic spindle, and the overlay of both corresponding fluorescent signals revealed a perfect co-localization (Fig. 2, lower panels, yellow staining), thus fully corroborating the above protein-protein interaction data.

### Cellular re-distribution of CLTA and chromosomal misalignment after MAD2B knockdown

**Figure 3:**
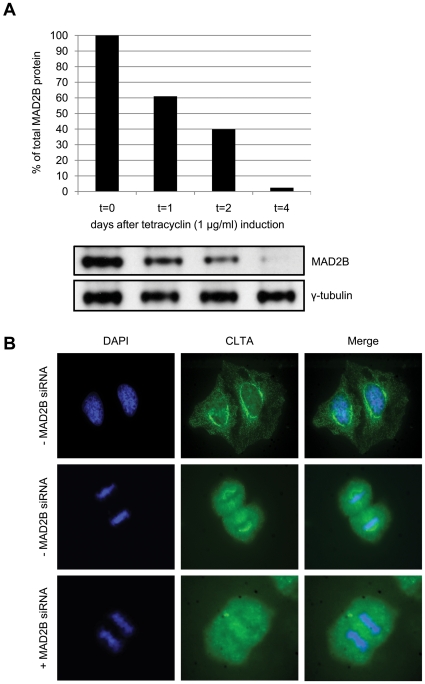
Cellular re-distribution of CLTA after MAD2B knockdown. (A) HEK293/T-REx cells were stably transfected with a pSUPERIOR-MAD2B siRNA construct (see Materials and methods). Subsequently, tetracyclin was added and cells were collected after 0, 1, 2 and 4 days, lysed and subjected to western blot analysis using anti-MAD2B and anti-γ-tubulin (control) antibodies. (B) HEK293/T-REx/pSUPERIOR-MAD2B cells were grown with or without tetracyclin (+/− MAD2B siRNA, respectively). Endogenous CLTA proteins were detected using an anti-CLTA antibody (green). DAPI staining (blue) was used to mark nuclei and chromosomes. In the presence of MAD2B CLTA localizes to mitotic spindles during mitosis, whereas after MAD2B depletion CLTA is re-distributed throughout the cell.

**Figure 4:**
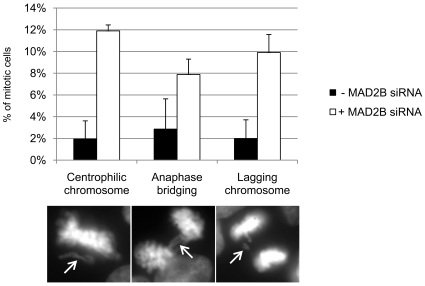
MAD2B depletion leads to mitotic defects. HEK293/T-REx/pSUPERIOR-MAD2B cells were transiently transfected with a H2B-RFP construct (see Materials and methods) and grown with or without tetracyclin (+/− MAD2B siRNA), respectively. Subsequently, mitotic cells were scored for misaligned chromosomes. Percentages of cells with misalignments, such as centrophilic chromosomes, anaphase bridges and lagging chromosomes, are shown in the histogram. Black bars represent cells normally expressing MAD2B (- MAD2B siRNA; see Fig. 3, t = 0), and white bars represent MAD2B depleted cells (+MAD2B siRNA; see Fig. 3, t = 4). In the lower panels examples of centrophilic chromosomes, anaphase bridges and lagging chromosomes are shown (arrows).

Having established that MAD2B and CLTA interact and co-localize at the spindle during mitosis, we next questioned what the effect on the sub-cellular localization of these proteins would be after depletion. To this end, we generated a stable MAD2B siRNA-inducible HEK293 cell line (see Materials and methods) in which, after 4 days of tetracyclin induction, the amount of MAD2B protein was reduced to 2% (Fig. 3A). Both non-depleted and MAD2B-depleted cells were fixed, incubated with an anti-CLTA antibody and analyzed by immunofluorescence. In accordance with the above exogenous expression data, we found that in the non-depleted cells endogenous CLTA also exhibited a punctuated cytoplasmic staining pattern with a perinuclear (Golgi) accumulation during interphase (Fig. 3B, upper panels). In MAD2B-depleted cells a similar pattern was observed (not shown). During mitosis endogenous CLTA was, again in accordance with the above exogenous expression data, localized at the mitotic spindle (Fig. 3B, middle panels). In contrast, however, we found that in the MAD2B-depleted cells endogenous CLTA no longer localized at the mitotic spindle but, instead, was re-distributed diffusely throughout the cell (Fig. 3B, lower panels). The endogenous localization of CLTC was unaffected in these cells (Fig. S2B). In order to assess the effect of MAD2B depletion on an orderly mitotic progression, we systematically analyzed mitotic figures from HEK293/T-REx MAD2B knock down cells (+MAD2B siRNA) using live cell imaging and compared these with those similarly obtained from wild-type HEK293/T-REx cells (- MAD2B siRNA). By doing so, we observed a significant overall increase (34.65% versus 11.65%, T-test, p = 0.030) in misaligned chromosomes in the knock down cells (Table S1), including centrophilic chromosomes, anaphase bridges and lagging chromosomes (Fig. 4; indicated by arrows in left, middle and right panels, respectively). A marked increase in chromosome aberrations after siRNA-mediated knock down of MAD2B has also been reported by Cheung et al. [19] in nasopharyngeal carcinoma cells in response to DNA damage, thus corroborating the results obtained here.

**Figure 5:**
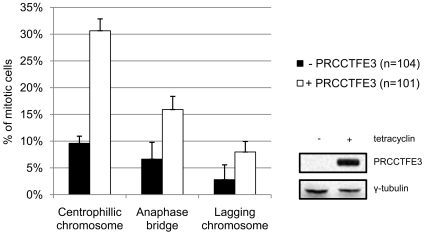
PRCCTFE3 expression leads to mitotic defects. HEK293/T-REx/PRCCTFE3 cells were transiently transfected with a H2B-RFP construct (see Materials and methods) and grown with or without tetracyclin (+/− PRCCTFE3), respectively. Subsequently, mitotic cells were scored for misaligned chromosomes. Percentages of cells with misalignments, such as centrophilic chromosomes, anaphase bridges and lagging chromosomes, are shown in the histogram. Black bars represent un-induced cells (- PRCCTFE3), and white bars represent induced cells (+PRCCTFE3). In the right panel, PRCCTFE3 protein expression in the induced (+ tetracyclin) cells is shown.

In order to assess whether exogenous expression of PRCCTFE3, which fails to shuttle MAD2B to the nucleus [15], has a similar effect on mitotic progression as siRNA-mediated MAD2B depletion, we systematically analyzed mitotic figures from induced HEK293/T-REx PRCCTFE3 cells (+PRCCTFE3) and compared these with those similarly obtained from un-induced HEK293/T-REx PRCCTFE3 cells (- PRCCTFE3). Also here, a significant overall increase (78.22% versus 30.10%, T-test, p = 0.024) in misaligned chromosomes was observed in the induced versus un-induced cells including, again, centrophilic chromosomes, anaphase bridges and lagging chromosomes (Fig. 5; Table S2).

## Discussion

Using a yeast two-hybrid-based interaction trap with MAD2B as a bait we identified the human clathrin light chain A (CLTA) as a novel *bona fide* interactor. The interaction was subsequently confirmed in mammalian cells using both *in vitro* and *in vivo* assays, and the interaction interface could be narrowed down to two domains, i.e., a C-terminal and a central domain. Previously, the CLTA C-terminal domain was found to function as a calmodulin-binding domain, whereas the central domain was found to function as a clathrin heavy chain (CLTC) binding domain [9]. This CLTC binding domain, in turn, was found to be essential for the formation of the clathrin trimeric structure, i.e., the clathrin triskelion [6]. It has been reported that the C-terminus of CLTA can also interact with the trimerization domain of the clathrin triskelion structure. We found that endogenous CLTC co-immunoprcipitates with MAD2B, suggesting that MAD2B may bind to clathrin in its triskelion conformation. Since our results additionally showed that full-length MAD2B protein is required for maintaining the interaction with CLTA, this interaction appears to be dependent on a proper folding of the protein and/or a proper exposure of its interaction interface(s) such as the HORMA domain [2], [20]. Additional evidence suggests that MAD2B may form multimeric configurations, a feature that has been extensively documented for the various protein-protein interactions exhibited by its closely related homolog MAD2 [21], [22]. Clathrin is similarly known to form multimeric lattices during clathrin-mediated membrane trafficking (i.e., coated vesicles) in interphase cells [23]. More recently it was discovered that clathrin may also play an important role in the mitotic spindle checkpoint [5]. Through gene trapping of nuclear proteins [24] and mass spectrometry of purified mitotic spindles [25] it has been established that clathrin is an integral component of the mitotic spindle. Using GFP-tagged clathrin light [18] and heavy [5] chains it was found that both subunits are recruited to microtubules as these invade the nuclear lamina during early prophase. Subsequently, clathrin remains associated with the spindle microtubules throughout chromosome congression, metaphase and chromosome segregation during anaphase. Only during telophase, clathrin is dissociated, followed by a re-establishment of clathrin proteins in the Golgi apparatus. Using immunofluorescence (electron) microscopy, it was found that clathrin is intimately associated with kinetochore fibers, bundles of microtubules that connect the spindle pole to the kinetochores of chromosomes [5]. When CLTC was depleted from the cells a number of mitotic defects was encountered, including misaligned chromosomes, such as centrophillic chromosomes, and destabilized kinetochore fibers. These defects were found to result in prolongation of mitosis due to continued signaling of the mitotic spindle checkpoint, presumably through MAD2 [5]. Since the N-terminal domain of CLTC binds to the spindle, it has been suggested that clathrin may act as a bridge between two or three microtubules within a spindle fiber to increase its stability [5].

MAD2B is, like its closely related homolog MAD2, a member of the MAD family of proteins [1], [2]. Whereas the role of MAD2 in mitotic spindle checkpoint control is well-documented, the exact role of MAD2B still remains to be established. We found that MAD2B physically interacts and co-localizes with CLTA at the mitotic spindle and that depletion of MAD2B from the cells leads to a redistribution of CLTA and a concomitant occurrence of mitotic defects. In addition, we found that upon MAD2B depletion the localization of CLTC to the mitotic spindle was unaffected. This latter observation is completely in line with data indicating that the determinant for spindle binding is contained within the heavy chain [5]. Based on our observations and those reported by others, we conclude that the MAD2B-CLTA interaction may be functional and, as such, may play a role in the control of an orderly mitotic progression. Whether a functional loss of this interaction induces destabilization of kinetochore fibres and a concomitant continued signalling of the mitotic spindle checkpoint still remains to be established.

Clathrin has been considered to be a ‘moonlighting protein’ which acts during interphase in membrane trafficking and during mitosis in stabilizing spindle fibers [9]. Other examples of such proteins include NUP107, NUP133 [26], NUP358 [27] and RANGAP1 [28]. These proteins are involved in nuclear transport during interphase and localize to kinetochores on the mitotic spindle during mitosis to ensure orderly mitotic progression [27]. Until recently, it was unclear which molecule(s) might recruit clathrin to the mitotic spindle. Yamauchi et al. [29]. reported that a B-MYB complex may ferry clathrin to the mitotic spindle Our results suggest that MAD2B may act to ferry the clathrin light chain CLTA to the mitotic spindle. In t(X;1)(p11;q21)-positive renal cell carcinomas expressing a PRCCTFE3 fusion protein this function may be abrogated through an impairment of the PRCC-MAD2B interaction and a concomitant failure to shuttle MAD2B to the nucleus [15]. Accordingly, our preliminary data show that exogenous expression of PRCCTFE3 has a deleterious effect on orderly mitotic progression reminiscent of that observed after siRNA enforced MAD2B depletion. Teleologically, this anomaly may contribute to tumor development analogous to what has been suggested for CLTC fusions with ALK in inflammatory myofibroblastic tumors and anaplastic large-cell lymphomas [10], and with TFE3 in renal cell carcinomas [11], [12]. Also these latter fusions are thought to disrupt clathrin trimerization, thereby impairing its function during mitosis [5].

## Materials and Methods

### Yeast two-hybrid assays

Yeast two-hybrid assays were performed essentially as described before using a hybriZAP human testis cDNA library containing approximately 4×10^6^ independent cDNA clones with an average insert size of ∼1 kb [15], [30]. Of these, 1×10^6^ clones were amplified once and 1×10^9^ of the resulting plaque forming units were mass-excised according to the manufacturer's instructions to generate an interaction cDNA library in pAD-GAL4. The yeast strain used for the interaction assays was pJ69-4A (a kind gift from Philip James). Positive interactions in this yeast strain can be selected for by adenine and histidine auxotrophy, next to β-galactosidase activity. The pJ69-4A strain was co-transfected with bait plasmid pBDT3C, carrying the human MAD2B coding sequence (AL031731), and the testis cDNA library using the Yeastmaker transformation kit (Clonetech). To select double transfectants, containing a pBD and a pAD vector, the transfected yeast cells were plated on synthetic defined (SD) medium lacking leucine and tryptophan (-LW). All resulting colonies were recovered from these plates, titrated, and re-plated on SD medium lacking histidine, adenine, leucine and tryptophane (-HALW) to reselect yeast clones expressing MAD2B interacting proteins. Colonies were allowed to grow for at least 5 days at 30°C after which replicate filters were lifted and tested for β-galactosidase activity according to the manufacturer's instructions (LacZ filter lift assay; Stratagene). The cDNA inserts of positive clones were isolated by direct PCR on yeast colonies with two pAD-GAL4 specific oligonucleotides, pAD-for (CTGTCACCTGGTTGGACGGACCAA) and pGAD-rev (GTGAACTTGCGGGGTTTTTCAG). All resulting PCR products were sequenced using the same oligonucleotides, and the pAD plasmids of these yeast clones were rescued in DH5α bacteria. Finally, the interacting cDNA clones were retransformed together with control plasmids as reported before [30]. Interactions were quantified by both a liquid β-galactosidase assay, using o-nitrophenyl-β-D-galactopyranoside (ONPG) as a substrate [31], and a liquid growth assay. To this end, pJ69-4A yeast cells were co-transfected with pAD plasmids carrying different CLTA (deletion) constructs (Fig. 1A) and the MAD2B bait plasmid. These co-transfectants were plated on SD-LW plates and incubated at 30°C for 3 days, after which three independent colonies were picked and grown to a stationary state in 5 ml of SD-LW medium (2 days at 30°C). Of these cultures, 15 µl was used to inoculate 15 ml of SD-HLW medium. During subsequent growth at 30°C the OD600 was monitored and, at the mid-log phase, the µmax was determined: µmax = [ln(xt) – ln(x0)]/t, where xt is the OD600 of the culture at t = t, x0 is the OD600 at t = 0 and t is the time between x0 and xt. The interaction scores for each CLTA deletion construct were expressed as means of three independent clones.

### Cloning and sequencing procedures

All cloning procedures were essentially as described before [17], [30], [32]. Sequence analyses were performed at the DNA sequence facility of the Radboud University Nijmegen Medical Center using a 3730 DNA Analyzer (Applied Biosystems). DNA and protein databases were searched using the BLAST or BLAT search algorithms at the NCBI or UCSC, respectively. Deletion constructs of human CLTA were generated by using 27–28 nt primers dispersed at intervals of 150 nt throughout the cDNA sequence. The 5′-end of the forward primers used were located at amino acid positions 1, 51, 101 and 151, respectively, according to BC_009201. The reverse primers were located at positions 218, 167 and 117. Deletion constructs of human MAD2B were generated using primers dispersed at intervals of 150 nt throughout the cDNA sequence as reported before [17]. The resulting PCR products were cloned into pGEM-T (Promega), sequence-verified, and subcloned into the correct pBDT3C and pGADT3C vectors.

### Cell culture

U2OS cells were cultured in DMEM (Invitrogen) supplemented with 10% FCS, penicillin (100 U/ml) and streptomycin (100 µg/ml) at 37°C and 7.5% CO_2_. HEK293/T-REx cells were cultured in DMEM supplemented with blasticidin (5 µg/ml), penicillin (100 U/ml) and streptomycin (100 µg/ml). HEK293/T-REx/PRCCTFE3 cells [15] were cultured in DMEM, supplemented with 10% FCS, zeocin (500 µg/ml), blasticidin (5 µg/ml), penicillin (100 units/ml) and streptomycin (100 µg/ml). For induction of PRCCTFE3 expression, tetracyclin was added to the culture medium (24 hours; 1 µg/ml). For the cellular localization studies, full length MAD2B was cloned into pDEST733 (mRFP; Invitrogen) and CLTA was cloned into pEGFP-N2 and pEGFP-C3 (Clontech). Cells were transiently transfected using an Amaxa Nucleofector according to the instructions of the manufacturer (Lonza). For localization of the proteins, cells were fixed one day after transfection for 30 min at room temperature with PBS containing 3.7% paraformaldehyde pH 7.4 and, subsequently, embedded in Vectashield with DAPI (Vector Labs). Cells were then analyzed by immunofluorescence and/or confocal laser scanning microscopy as reported before [30]. For live cell imaging, cells were transiently transfected with a histone 2B containing pTSIN- H2B-RFP construct (a kind gift from Jan van Deursen) and scored for chromosome misalignments during mitosis using a Zeiss Axiovert 200 M microscope. Cells were synchronized using a double thymidine block (early S-phase block). To this end, cells were incubated in DMEM supplemented with thymidine (2 mM) for 16 h, washed three times, incubated in complete DMEM without thymidine and grown for another 8 h prior to a second incubation with thymidine (2 mM) for 16 h. After release from this second block, cells were grown in complete DMEM during several time intervals. During these time intervals, cell cycle stages were determined by FACS analysis as reported before [17], [32], [33]. For ^35^S methionine labelling, cells were incubated for 30 min in DMEM minus methionine (Invitrogen) supplemented with 10% FCS, penicillin (100 U/ml) and streptomycin (100 µg/ml) at 37°C and 7.5% CO_2_, followed by removal of the medium and adding DMEM minus methionine supplemented with 10% FCS, penicillin (100 U/ml), streptomycin (100 µg/ml) and 0.25 mCi ^35^S methionine. Cells were incubated for 16 h at 37°C and 7.5% CO_2_ before harvesting.

### GST pull-down and co-immunoprecipitation assays

The full length CLTA coding sequence was cloned into a pDEST15 vector (Invitrogen) and transformed into BL21-DE3 cells (Promega), yielding a fusion protein with a GST tag at the N-terminus (GST-CLTA). Purification of this fusion protein was performed as described by Kantardzhieva et al. [34] **.** The full length MAD2B coding sequence was cloned into a pDEST303 vector, yielding a HA-tagged MAD2B protein (HA-MAD2B) and transfected into U2OS cells after sequence verification. For the GST pull-down assays, 50 µl of GST-CLTA was combined with 100 µl of lysates from U2OS cells expressing HA-MAD2B. In addition, 50 µl glutathione Sepharose 4B slurry (Amersham Biosciences) was used. A protease inhibitor cocktail (Roche) was added during all wash steps. Before use, the sepharose beads were washed twice with TBS (25 mM Tris pH 7.4 and 150 mM NaCl), followed by a 2 h incubation of the GST-CLTA (and GST alone as a control) with the beads. After this incubation, the GST-coupled beads were washed with TBSTD buffer (25 mM Tris pH 7.4, 150 mM NaCl, 1.0% Triton X-100 and 2 mM DTT), and incubated overnight at 4°C with lysates of U2OS cells expressing HA-MAD2B (see above). After five additional washes with lysis buffer (50 mM Tris-HCl pH 7.5, 150 mM NaCl and 0.5% Triton X-100) and washing buffer (50 mM Tris-HCl pH 7.5, 100 mM NaCl, 2 mM MgCl_2_, 2 mM CaCl_2_, 1% Triton X-100 and 2 mM DTT) the samples were denatured and loaded onto 4–12% NuPAGE polyacrylamide gels (Invitrogen). After electrophoreses the gels were blotted onto nitrocellulose membranes (Protran, Schleicher and Schuell). The resulting blots were blocked in PBS with 5% non fat dry milk and incubated with anti-MAD2B antibody for 1 h at room temperature and, subsequently, with either a peroxidase conjugated secondary antibody (Zymed) or a fluorescent conjugated secondary antibody (Molecular Probes). Peroxidase signals were visualized using auto-radiographic exposure to Kodak X-Omat films, while fluorescent signals were scanned and analyzed with the Odyssey system and its associated software (Li-Cor). For immunoprecipitation studies, the full length CLTA coding sequence was cloned into a pDEST306 vector, yielding a FLAG-tagged CLTA protein (FLAG-CLTA) and transfected into U2OS cells after sequence verification. Cells were washed three times in ice-cold PBS and scraped from the tissue culture dishes. Total cell lysates were prepared in 1 ml lysis buffer (PBS,10% glycerol, 0.1% NP40, protease inhibitors), kept on ice for 10 min while vortexed gently at 2 min intervals, and centrifuged for 30 min at 38.500 rpm at 4°C. The supernatants were pre-cleared in 100 µl of protein A/G PLUS Sepharose (50% slurry in lysis buffer; Santa Cruz) for 1 h at 4°C and subsequently centrifuged for 3 min at 2500 rpm at 4°C. Immunoprecipitations were performed with these pre-cleared supernatants by adding 1–5 µg primary antibody, followed by a 1 h rotation at 4°C. Subsequently, 25 µl protein A/G PLUS Sepharose (50% slurry in lysis buffer; Santa Cruz) was added to the immune complexes for 1 h at 4°C. The sepharose-bound immune complexes were collected by centrifugation (3 min; 2500 rpm; 4°C) and the resulting pellets were washed five times with lysis buffer before antigens were released by heating at 95°C for 5 min. Western blotting was performed as described above. For quantification of western blot signals the ImageJ software tool was used (http://rsb.info.nih.gov/ij/). The area under the curve (AUC) of a specific signal was corrected by the AUC of the loading control. The measurements of uninduced samples were arbitrarily set at 100% and the other conditions were recalculated correspondingly, thereby allowing ratio comparisons.

### RNA interference assays

MAD2B-specific siRNA constructs were generated in a pSUPERIOR vector (OligoEngine). To this end, the following primers were used: forward GATCCCCCTGTGAGTTGTTTCAATAAttcaagagaTTA-TTGAAACAACTCACAGTTTTTGGAAA and reverse AGCTTTTCCAAAAACTGTGAGTTGTTTCAATA-AtctcttgaaTTATTGAAACAACTCACAGGGG. The forward and reverse strands of oligo's containing the MAD2B siRNA-expressing sequence were annealed and subsequently cloned into pSUPERIOR. Two days after transfection of this construct into HEK293/T-REx cells, transfected cells were selected using 2 µg/ml puromycin (Sigma Aldrich), expanded and tested for MAD2B expression after tetracyclin induction.

### Antibodies

A polyclonal rabbit anti-MAD2B antibody was used as described before [15]. Monoclonal mouse anti-FLAG (Sigma), monoclonal mouse anti-HA (Sigma), monoclonal mouse anti-γ-tubulin (Abcam), polyclonal rabbit anti-α-tubulin (Santa Cruz), monoclonal mouse anti-CLTA (Sigma), polyclonal rabbit anti-CLTA (Protein Tech Group) and monoclonal mouse anti-CLTC (*alias* CHC; Abcam) antibodies were used according to the instructions of the manufacturers.

## Supporting Information

Figure S1 **Synchronization of U2OS cells and cell cycle distribution.** U2OS cells were synchronized using a double thymidine block (early S-phase block; see Materials and Methods) and subsequently released during several time intervals (t = 0 to t = 15 h). Concurrent cell cycle distributions were measured using FACS analysis and expressed as percentages G0/G1, S and G2/M cells, respectively [31]; [32].
(PDF)

Figure S2 **CLTC localization is not affected by MAD2B knockdown.** (A) U2OS cells were grown in the presence of ^35^S-labelled methionine. Subsequent immunoprecipitations were performed on cell lysates using anti-CLTA, anti-MAD2B and control (IgG) antibodies. CLTC marks the position of the clathrin heavy chain. (B) HEK293/T-REx/pSUPERIOR-MAD2B cells were grown in the presence of tetracyclin (+MAD2B siRNA). Endogenous CLTC proteins were detected using an anti-CLTC antibody (red). DAPI staining (blue) was used to mark nuclei and chromosomes. CLTC localizes to the mitotic spindle during mitosis after MAD2B depletion.
(PDF)

Figure S3 **CLTA co-localizes with α-tubulin at the mitotic spindle.** U2OS cells were stained with anti-CLTA (green) and anti-α-tubulin (red) antibodies. DAPI staining (blue) marks the position of the chromosomes. Yellow staining in the overlay indicates co-localization of endogenous CLTA and α-tubulin at the mitotic spindle.
(PDF)

Table S1 **Mitotic defects in HEK293/T-REx after MAD2B depletion.** HEK293/T-REx/pSUPERIOR-MAD2B cells were transiently transfected with a H2B-RFP construct (see Materials and methods) and grown with or without of tetracyclin (+/− MAD2B siRNA), respectively. Subsequently, cells were live recorded and scored for chromosome misalignments during mitosis. Numbers (#) and percentages (%) of cells with misalignments, such as centrophilic chromosomes, anaphase bridges and lagging chromosomes are listed.
(DOC)

Table S2 **Mitotic defects in HEK293/T-REx after PRCCTFE3 induction.** HEK293/T-REx/PRCCTFE3 cells were transiently transfected with a H2B-RFP construct (see Materials and methods) and grown with or without of tetracyclin (+/− PRCCTFE3), respectively. Subsequently, cells were live recorded and scored for chromosome misalignments during mitosis. Numbers (#) and percentages (%) of cells with misalignments, such as centrophilic chromosomes, anaphase bridges and lagging chromosomes are listed.
(DOC)
