# Supplementary figures and images for: The Mitotic Arrest Deficient Protein MAD2B Interacts with the Clathrin Light Chain A during Mitosis
Source: PLoS One. 2010 Nov 30;5(11):e15128. doi: 10.1371/journal.pone.0015128 (PMC2994903; doi:10.1371/journal.pone.0015128)

unsynchronized

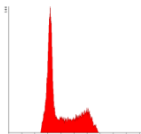

|       |     |
|-------|-----|
| G0/G1 | 59% |
| S     | 20% |
| G2/M  | 21% |

t = 0

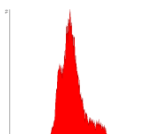

|       |     |
|-------|-----|
| G0/G1 | 13% |
| S     | 72% |
| G2/M  | 15% |

t = 3

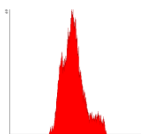

|       |     |
|-------|-----|
| G0/G1 | 10% |
| S     | 69% |
| G2/M  | 21% |

t = 6

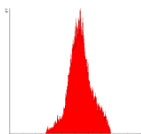

|       |     |
|-------|-----|
| G0/G1 | 3%  |
| S     | 48% |
| G2/M  | 49% |

t = 9

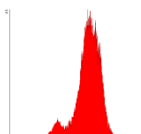

|       |     |
|-------|-----|
| G0/G1 | 3%  |
| S     | 12% |
| G2/M  | 85% |

t = 12

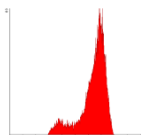

|       |     |
|-------|-----|
| G0/G1 | 7%  |
| S     | 12% |
| G2/M  | 81% |

t = 15

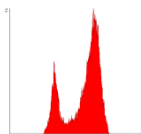

|       |     |
|-------|-----|
| G0/G1 | 19% |
| S     | 12% |
| G2/M  | 69% |

Supplement: Figure S1 — Synchronization of U2OS cells and cell cycle distribution. U2OS cells were synchronized using a double thymidine block (early S-phase block; see Materials and Methods) and subsequently released during several time intervals (t = 0 to t = 15 h). Concurrent cell cycle distributions were measured using FACS analysis and expressed as percentages G0/G1, S and G2/M cells, respectively [31]; [32]. (PDF) [file pone.0015128.s001.pdf]

**A**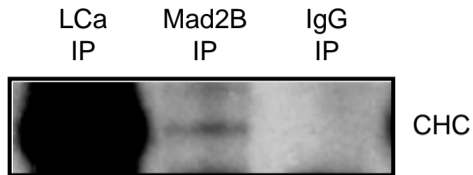**B**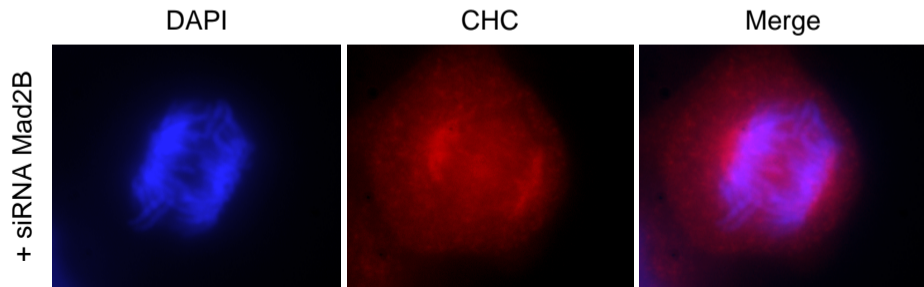

Supplement: Figure S2 — CLTC localization is not affected by MAD2B knockdown. (A) U2OS cells were grown in the presence of 35S-labelled methionine. Subsequent immunoprecipitations were performed on cell lysates using anti-CLTA, anti-MAD2B and control (IgG) antibodies. CLTC marks the position of the clathrin heavy chain. (B) HEK293/T-REx/pSUPERIOR-MAD2B cells were grown in the presence of tetracyclin (+MAD2B siRNA). Endogenous CLTC proteins were detected using an anti-CLTC antibody (red). DAPI staining (blue) was used to mark nuclei and chromosomes. CLTC localizes to the mitotic spindle during mitosis after MAD2B depletion. (PDF) [file pone.0015128.s002.pdf]

DAPI

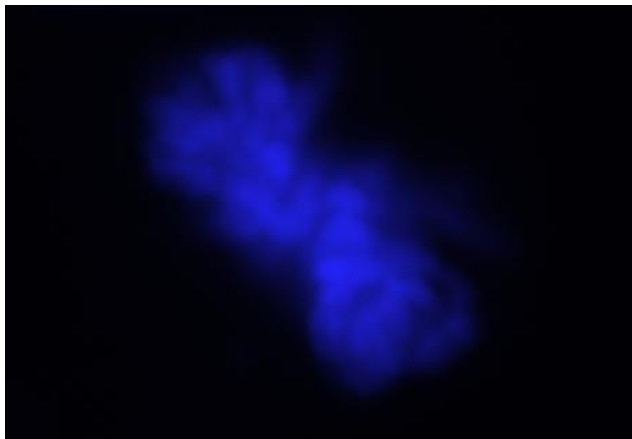

Merge

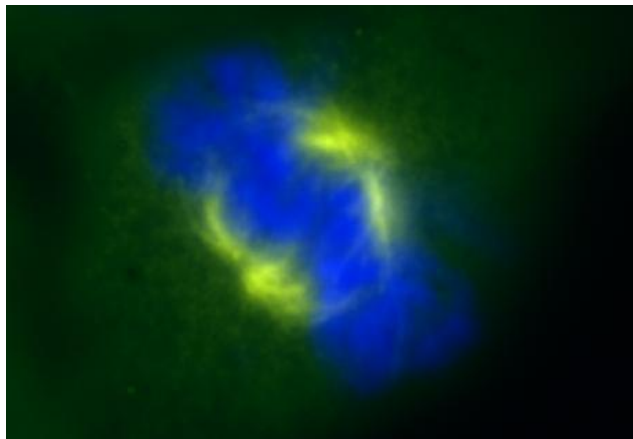

LCa

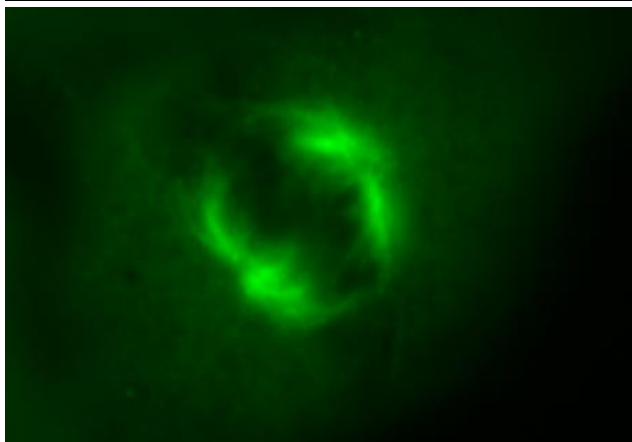

$\alpha$ -tubulin

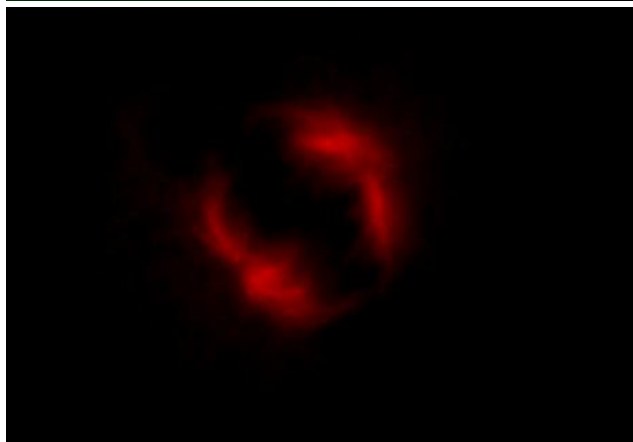

Supplement: Figure S3 — CLTA co-localizes with α-tubulin at the mitotic spindle. U2OS cells were stained with anti-CLTA (green) and anti-α-tubulin (red) antibodies. DAPI staining (blue) marks the position of the chromosomes. Yellow staining in the overlay indicates co-localization of endogenous CLTA and α-tubulin at the mitotic spindle. (PDF) [file pone.0015128.s003.pdf]
