# Supplementary material for: The Mitotic Arrest Deficient Protein MAD2B Interacts with the Clathrin Light Chain A during Mitosis
Source: PLoS One. 2010 Nov 30;5(11):e15128. doi: 10.1371/journal.pone.0015128 (PMC2994903; doi:10.1371/journal.pone.0015128)
Supplement: Table S1 — Mitotic defects in HEK293/T-REx after MAD2B depletion. HEK293/T-REx/pSUPERIOR-MAD2B cells were transiently transfected with a H2B-RFP construct (see Materials and methods) and grown with or without of tetracyclin (+/− MAD2B siRNA), respectively. Subsequently, cells were live recorded and scored for chromosome misalignments during mitosis. Numbers (#) and percentages (%) of cells with misalignments, such as centrophilic chromosomes, anaphase bridges and lagging chromosomes are listed. (DOC) [file pone.0015128.s004.doc]

|  | ***+ MAD2B siRNA*** | | ***- MAD2B siRNA*** | |
| --- | --- | --- | --- | --- |
|  | ***# of cells*** | ***% of cells*** | ***# of cells*** | ***% of cells*** |
| Tripolar segregation | 6 | 5,94% | 5 | 4,85% |
| Centrophillic chromosome | 12 | 11,88% | 2 | 1,94% |
| Anaphase bridging | 8 | 7,92% | 3 | 2,91% |
| Lagging chromosome | 9 | 8,91% | 2 | 1,94% |
|  |  |  |  |  |
| Total abnormal mitotic figures | 35 | 34,65% | 12 | 11,65% |
| Normal mitotic figures | 66 | 65,35% | 91 | 88,35% |
|  |  |  |  |  |
| *Total* | 101 | 100% | 103 | 100% |

**Table S1. Mitotic defects in HEK293 T-REx after MAD2B depletion.** HEK293/T-REx/pSUPERIOR-MAD2B cells were transiently transfected with a H2B-RFP construct (see Materials and methods) and grown with or without of tetracyclin (+ / - MAD2B siRNA), respectively. Subsequently, cells were live recorded and scored for chromosome misalignments during mitosis. Numbers (#) and percentages (%) of cells with misalignments, such as centrophilic chromosomes, anaphase bridges and lagging chromosomes are listed.
