# Supplementary material for: The Mitotic Arrest Deficient Protein MAD2B Interacts with the Clathrin Light Chain A during Mitosis
Source: PLoS One. 2010 Nov 30;5(11):e15128. doi: 10.1371/journal.pone.0015128 (PMC2994903; doi:10.1371/journal.pone.0015128)
Supplement: Table S2 — Mitotic defects in HEK293/T-REx after PRCCTFE3 induction. HEK293/T-REx/PRCCTFE3 cells were transiently transfected with a H2B-RFP construct (see Materials and methods) and grown with or without of tetracyclin (+/− PRCCTFE3), respectively. Subsequently, cells were live recorded and scored for chromosome misalignments during mitosis. Numbers (#) and percentages (%) of cells with misalignments, such as centrophilic chromosomes, anaphase bridges and lagging chromosomes are listed. (DOC) [file pone.0015128.s005.doc]

|  | ***+ PRCCTFE3*** | | ***- PRCCTFE3*** | |
| --- | --- | --- | --- | --- |
|  | ***# of cells*** | ***% of cells*** | ***# of cells*** | ***% of cells*** |
| Tripolar segregation | 24 | 23,76% | 11 | 10,68% |
| Centrophillic chromosome | 31 | 30,69% | 10 | 9,71% |
| Anaphase bridging | 16 | 15,84% | 7 | 6,80% |
| Lagging chromosome | 8 | 7,92% | 3 | 2,91% |
|  |  |  |  |  |
| Total abnormal mitotic figures | 79 | 78,22% | 31 | 30,10% |
| Normal mitotic figures | 25 | 24,75% | 70 | 67,96% |
|  |  |  |  |  |
| *Total* | 104 | 100% | 101 | 100% |

**Table S2. Mitotic defects in HEK293 T-REx after PRCCTFE3 induction.** HEK293/T-REx/PRCCTFE3 cells were transiently transfected with a H2B-RFP construct (see Materials and methods) and grown with or without of tetracyclin (+ / - PRCCTFE3), respectively. Subsequently, cells were live recorded and scored for chromosome misalignments during mitosis. Numbers (#) and percentages (%) of cells with misalignments, such as centrophilic chromosomes, anaphase bridges and lagging chromosomes are listed.
